# Tumor size is an independent negative prognostic factor for event free survival in children with differentiated thyroid cancer

**DOI:** 10.3389/fendo.2022.979054

**Published:** 2022-08-25

**Authors:** Sandeep Kumar Parvathareddy, Abdul K. Siraj, Padmanaban Annaiyappanaidu, Nabil Siraj, Wael Haqawi, Saif S. Al-Sobhi, Fouad Al-Dayel, Khawla S. Al-Kuraya

**Affiliations:** ^1^ Human Cancer Genomic Research, Research Center, King Faisal Specialist Hospital and Research Centre, Riyadh, Saudi Arabia; ^2^ Department of Surgery, King Faisal Specialist Hospital and Research Centre, Riyadh, Saudi Arabia; ^3^ Department of Pathology, King Faisal Specialist Hospital and Research Centre, Riyadh, Saudi Arabia

**Keywords:** differentiated thyroid carcinoma, children, tumor size, event-free survival, distant metastasis

## Abstract

**Background:**

The incidence of pediatric differentiated thyroid carcinoma (DTC) is increasing. Despite the advanced disease at presentation, the overall prognosis of DTC in children is excellent. The aim of this study is to investigate the risk stratifying factors for event free survival (EFS) of pediatric DTC from Middle Eastern ethnicity.

**Methods:**

Eighty-eight patients aged ≤18 years with diagnosis of primary DTC were retrospectively analyzed. Cox proportional hazards model were used to calculate Hazard Ratios (HR) and Kaplan–Meier analysis were conducted to investigate EFS.

**Results:**

Eighty-eight (23 males and 65 females) pediatric DTCs who underwent surgery and radioactive iodine therapy had been reported (median age at diagnosis 15 years; range 5.9-17.9), with lymph node metastasis (LNM) noted in 70.5% and distant metastasis in 13.6%. Mean follow-up was 8.4 years. Ten-year overall survival rate was 98.4% while 10-year EFS was 79.2%. EFS was negatively impacted by the presence of LNM, distant metastasis and tumor size >4cm. American Thyroid Association risk stratification did not impact EFS in our cohort. Multivariate analysis revealed tumor size >4cm (HR = 5.34; 95% confidence interval (CI) = 1.36 – 20.22; p = 0.0177) and distant metastasis (HR = 8.73; 95% CI = 1.48 – 60.05; p = 0.0154) as independent negative prognostic factors for EFS.

**Conclusions:**

Primary tumor size and the presence of distant metastasis at diagnosis are the only independent prognostic risk factors for EFS in pediatric DTC in Middle Eastern ethnicity. Children with tumor size over 4cm had poor EFS, which may justify the need of more aggressive treatment and frequent follow-up.

## 1 Introduction

Differentiated thyroid carcinomas (DTC) are the most common endocrine malignancy, and account for more than 4% of all pediatric cancers (1). Papillary Thyroid Carcinoma (PTC) accounts for approximately 90% of pediatric DTC, whereas follicular thyroid carcinoma (FTC) accounts for about 10% (2, 3). The incidence of pediatric DTC has been increasing over the decades (4–6). Pediatric DTC differ clinically from adult DTC. At diagnosis children usually present with more advanced disease, with larger tumor size, frequent extrathyroidal extension (ETE), and high rate of lymph node metastasis and distant metastasis (7–9). Molecular differences between pediatric and adult DTC have also been established. *BRAF* mutation is not as common a driver mutation as in adult DTC and RAS gene mutations are rare (7, 10, 11). These molecular differences could contribute to the difference in clinical behavior between adult and pediatric DTC.

Despite the aggressive behavior, the prognosis of pediatric DTC is excellent (12, 13). The management of DTC in children can be challenging, given the excellent prognosis and the extremely low disease specific mortality, regardless of the presence of metastasis. Knowledge regarding prognostic factors for survival in pediatric patients with DTC, can be helpful for individualized therapy and follow-up. In 2015, the American Thyroid Association (ATA) published management guidelines for children with DTC, where these guidelines classify patient into low, intermediate and high risk categories (14). Recent studies have illustrated that ATA risk group is a prognostic factor for event-free survival (EFS) in pediatric DTC patients (8, 15, 16).

The ultimate aim of this retrospective study was to determine the prognostic factors for EFS in pediatric DTC from Middle Eastern ethnicity and whether ATA risk classification is a predictor of recurrence in pediatric DTC.

## 2 Materials and methods

### 2.1 Patient selection

Eighty-eight pediatric (≤ 18 years) DTC patients diagnosed between 1988 and 2018 at King Faisal Specialist Hospital and Research Centre (Riyadh, Saudi Arabia) who underwent surgery and radioactive iodine (RAI) therapy were included in the study. Cases were identified based on clinical history followed by fine needle aspiration biopsy for confirmation. The Institutional Review Board of the hospital approved this study and since only retrospective patient data were used, the Research Advisory Council (RAC) provided waiver of consent under project RAC # 221 1168 and # 2110 031. The study was conducted in accordance with Declaration of Helsinki.

### 2.2 Clinico-pathological and follow-up data

Baseline clinico-pathological data were collected from case records and have been summarized in **Table 1**. Of the 88 patients included in our study, only 2.3% (2/88) underwent lobectomy/hemithyroidectomy alone, whereas 5.7% (5/88) of patients underwent total thyroidectomy alone and 92.0% (81/88) underwent thyroidectomy with lymph node dissection (prophylactic central lymphadenectomy was done in 15.9% (14/88) and therapeutic central/lateral lymphadenectomy in 76.1% (67/88)). Extrathyroidal extension (ETE) was further classified as follows: microscopic ETE was defined as tumor extending beyond the thyroid capsule into the surrounding peri-thyroidal soft tissues of fat and/or skeletal muscle, without visual evidence of this invasion and macroscopic ETE defined as visual evidence of tumor invasion into strap muscles, subcutaneous soft tissue, larynx, trachea, esophagus, recurrent laryngeal nerve or prevertebral fascia. Staging of DTC was performed using the eighth edition of American Joint Committee on Cancer (AJCC) staging system. All the patients included in this study had undergone RAI therapy with an average dosage of 186 mCi. 34.1% (30/88) of patients had undergone multiple treatments with RAI (16 patients received two doses, 11 received three doses, two received four doses and one patient received five doses) and the average dosage for these patients was 328 mCi. Patients were stratified into low, intermediate and high risk based on 2015 American Thyroid Association (ATA) guidelines (17). Following initial surgery, low-risk DTC patients were followed up annually, intermediate risk patients were followed up at 6 months’ intervals and high risk patients were followed up at 3 months’ intervals. At each follow-up, neck ultrasound, thyroid function tests, thyroglobulin levels and thyroglobulin antibodies were performed. In addition, for high risk patients, radioiodine scan and PET CT scan were performed to identify tumor recurrence. Complete remission was defined as serum thyroglobulin (Tg) levels below the reference range and no evidence of structural disease. Partial response was defined as decreasing Tg levels and/or functional evidence of disease after surgery and RAI therapy. Persistent disease was defined as biochemical, structural or functional evidence of disease within one year of surgery. Recurrence was defined as any new biochemical or structural disease following complete remission. Response to therapy was evaluated based on criteria adapted from Tuttle et al. (18, 19), whereby excellent response was defined as complete remission achieved during the first two years of follow-up and all other cases being classified as poor response.

**Table 1:** Clinico-pathological characteristics of pediatric differentiated thyroid carcinoma who underwent surgery and RAI therapy.

	No.	%
**Total**	88	
**Median age (range)**	15.0 (5.9 – 17.9)	
≤15	49	55.7
>15	39	44.3
**Gender**		
Female	65	73.9
Male	23	26.1
**Histologic subtype**		
PTC	84	95.5
Classical variant	55	62.5
Follicular variant	10	11.4
Tall cell variant	6	6.8
Other variants	13	14.8
FTC	4	4.5
**Tumor laterality**		
Unilateral	54	61.4
Bilateral	34	38.6
**Multifocality**		
Yes	51	58.0
No	37	42.0
**Extrathyroidal extension**		
Gross	8	9.0
Microscopic	40	45.5
Absent	40	45.5
**Lymphovascular invasion**		
Present	44	50.0
Absent	44	50.0
**Tumor size**		
≤4cm	65	73.9
>4cm	23	26.1
**Lymph node metastasis**		
No	19	21.6
Yes	62	70.5
Unknown	7	7.9
**Number of lymph nodes dissected**		
Median (range)	20 (2 – 68)	
**Number of positive lymph nodes**		
Median (range)	7 (0 – 31)	
**Distant metastasis**		
Yes	12	13.6
No	76	86.4
**TNM Stage**		
I	83	94.3
II	5	5.7
**Persistent disease**		
Yes	12	13.6
No	76	86.4
**Recurrence**		
Yes	22	25.0
No	66	75.0
**Response to therapy**		
Excellent	71	80.7
Poor	17	19.3
**Number of surgeries**		
One	54	61.4
Two	25	28.4
Three	9	10.2
**ATA risk category**		
Low	11	12.5
Intermediate	32	36.4
High	45	51.1
**Post-operative thyroglobulin**		
Positive	32	38.1
Negative	52	61.9
**Hashimoto’s thyroiditis**		
Yes	24	27.3
No	64	72.7
**Status**		
Alive	87	98.9
Dead due to PTC	0	0.0
Dead due to other causes	1	1.1
***BRAF* mutation**		
Present	18	20.5
Absent	54	61.4
Unknown	16	18.1
***TERT* mutation**		
Present	0	0.0
Absent	76	86.4
Unknown	12	13.6

### 2.3 *BRAF* and *TERT* mutation analysis

*BRAF* and *TERT* mutation data for the DTC cohort was available from our previous studies (20, 21).

### 2.4 Statistical analysis

The associations between clinico-pathological variables and patient age was performed using contingency table analysis and Chi square tests. Overall survival (OS) and event-free survival (EFS) were determined using Kaplan-Meier estimates. OS was defined as the time from diagnosis to death from any cause. EFS was defined as the time from diagnosis to the occurrence of persistent or recurrent disease. Cox proportional hazards model was used for analyzing the impact of prognostic factors on EFS in univariate and multivariate manner. Two-sided tests were used for statistical analyses with a limit of significance defined as p value < 0.05. Data analyses were performed using the JMP14.0 (SAS Institute, Inc., Cary, NC) software package.

## 3 Results

### 3.1 Patient and tumor characteristics

Median age of the entire cohort was 15 years (range: 5.9 – 17.9 years), with a male: female ratio of 1:3. Majority of the tumors were PTC (95.5%; 84/88). Regional lymph node metastasis (LNM) was noted in 70.5% (62/88) of cases and distant metastasis was present in 13.6% (12/88), of which synchronous distant metastasis was noted in 8.0% (7/88) and metachronous distant metastasis was noted in 5.6% (5/88). The site of distant metastasis in all 12 patients was the lung. Mean follow-up was 8.4 years (range 3.1 – 25.7 years). Ten-year overall survival and event-free survival (EFS) rates were 98.4% and 79.2%. Events occurred in 32.9% (29/88), including locoregional recurrence (n = 12), pulmonary metastasis (n = 5) and persistent disease (n = 12) (**Table 1**). Of the 12 patients with persistent disease, five had biochemical persistence and seven had structural persistence. Among the seven patients with structural persistence, three had lymph node persistence and four had distant persistent disease.

To determine the optimal tumor diameter cut-off for analysis, we divided the tumor sizes into four groups (<2cm, 2-3cm, 3-4cm and >4cm) and analysed the EFS for these groups. On both univariate and multivariate analysis, we found that tumor size more than 4cm was the most robust predictor of EFS (**Table 2**). 26.1% (23/88) of DTCs in our cohort were larger than 4cm in largest diameter.

**Table 2:** The Association between tumor size and event-free survival in pediatric differentiated thyroid carcinoma.

Tumor size	Event-free survival			
	Unadjusted		Adjusted*	
	HR (95% CI)	p value	HR (95% CI)	p value
<2cm	Reference		Reference	
2-3cm	0.86 (0.18 – 3.13)	0.8248	0.72 (0.12 – 3.55)	0.6862
3-4cm	1.50 (0.44 – 4.78)	0.4992	2.16 (0.56 – 8.26)	0.2552
>4cm	3.17 (1.28 – 8.62)	0.0126	5.01 (1.53 – 18.65)	0.0073

*Adjusted for age, gender, laterality, focality, extrathyroidal extension, lymphovascular invasion, regional lymph node metastasis, synchronous distant metastasis.

HR, Hazard ratio; CI, Confidence interval.

### 3.2 Factors predicting event-free survival

On univariate analysis, EFS was impaired for patients with gross extrathyroidal extension (p = 0.0229), tumor size larger than 4cm (p = 0.0245), regional lymph node metastasis (p = 0.0132), synchronous distant metastasis (p = 0.0055) and response to therapy (p = 0.0436). However, on multivariate analysis, only tumor size larger than 4cm (Hazard ratio = 5.34; 95% confidence interval = 1.36 – 20.22; p = 0.0177) and synchronous distant metastasis (Hazard ratio = 8.73; 95% confidence interval = 1.48 – 60.05; p = 0.0154) were found to be independent negative predictors of EFS (**Table 3**).

**Table 3:** Cox proportional hazards model for predictors of event-free survival in pediatric differentiated thyroid carcinoma.

	Event-free survival			
	Univariate		Multivariate	
Clinico-pathological variables	HR (95% CI)	p value	HR (95% CI)	p value
**Age** ≤ 15 years (vs. > 15 years)	2.22 (0.91 – 6.21)	0.0816		
**Gender** Male (vs. Female)	0.31 (0.07 – 0.93)	0.0354	0.16 (0.03 – 0.56)	0.0027
**Tumor focality** Multifocal (vs. unifocal)	0.94 (0.40 – 2.30)	0.8919		
**Tumor laterality** Bilateral (vs. Unilateral)	1.55 (0.66 – 3.65)	0.3092		
**Extrathyroidal extension** Absent Microscopic Gross	Reference 2.00 (0.77 – 5.76) 4.68 (1.18 – 16.61)	0.1550 0.0229	Reference 1.35 (0.27 – 6.41) 0.70 (0.11 – 4.03)	0.7109 0.6885
**Lymphovascular invasion** Present (vs. Absent)	1.06 (0.42 – 2.55)	0.8934		
**Tumor size** >4cm (vs. ≤4cm)	2.73 (1.14 – 6.36)	0.0245	5.34 (1.36 – 20.22)	0.0177
**Regional LN metastasis** Present (vs. absent)	6.61 (1.38 – 118.70)	0.0132	1.89 (0.32 – 36.03)	0.5275
**Synchronous distant metastasis** Present (vs. absent)	3.85 (1.53 – 9.02)	0.0055	8.73 (1.48 – 60.05)	0.0154
**ATA risk category** Low Intermediate High	Reference - -	0.0343 0.0036	Reference - -	0.0603 0.1246
**Response to therapy** Poor (vs. excellent)	2.50 (1.03 – 5.82)	0.0436	0.63 (0.12 – 3.21)	0.5832
**BRAF mutation** Present (vs. absent)	0.55 (0.13 – 1.64)	0.3050		

HR, Hazard ratio; CI, Confidence interval; LN, Lymph node.

### 3.3 Age and clinico-pathological associations

Since our data showed a trend towards association between age (15 years) and EFS (Hazard ratio = 2.22; 95% confidence interval = 0.91 – 6.21; p = 0.0816; **Table 3**), we sought to determine the clinico-pathological associations with age. Since the median age of our cohort was 15 years, we divided the patients based on this age cut-off. Younger age (≤ 15 years) was associated with adverse clinico-pathological parameters such as male gender (p = 0.0368), tumor larger than 4cm (p = 0.0368), distant metastasis (p = 0.0295) and poor response to therapy (p = 0.0102) (**Table 4**).

**Table 4:** Clinico-pathological characteristics of pediatric differentiated thyroid carcinoma who underwent surgery and RAI therapy.

	Total		Age ≤ 15 years		Age > 15 years		
	No.	%	No.	%	No.	%	p value
**Total**	88		49	55.7	39	44.3	
**Gender**							
Female	65	73.9	32	65.3	33	84.6	0.0368
Male	23	26.1	17	34.7	6	15.4	
**Histologic subtype**							
PTC	84	95.5	47	95.9	37	94.9	0.8154
FTC	4	4.5	2	4.1	2	5.1	
**Tumor laterality**							
Unilateral	54	61.4	26	53.1	28	71.8	0.0709
Bilateral	34	38.6	23	46.9	11	28.2	
**Multifocality**							
Yes	51	58.0	30	61.2	21	53.8	0.4863
No	37	42.0	19	38.8	18	46.2	
**Extrathyroidal extension**							
Gross	8	9.0	5	10.2	3	7.7	0.1806
Microscopic	40	45.5	26	53.1	14	35.9	
Absent	40	45.5	18	36.7	22	56.4	
**Lymphovascular invasion**							
Present	44	50.0	24	49.0	20	51.3	0.8301
Absent	44	50.0	25	51.0	19	48.7	
**Tumor size**							
≤4cm	65	73.9	32	65.3	33	84.6	0.0368
>4cm	23	26.1	17	34.7	6	15.4	
**Lymph node metastasis**							
No	19	23.5	7	15.9	12	32.4	0.0801
Yes	62	76.5	37	84.1	25	67.6	
**Distant metastasis**							
Yes	12	13.6	10	20.4	2	5.1	0.0295
No	76	86.4	39	79.6	37	94.9	
**TNM Stage**							
I	83	94.3	46	93.9	37	94.9	0.8408
II	5	5.7	3	6.1	2	5.1	
**Recurrence**							
Yes	22	25.0	16	32.7	6	15.4	0.0586
No	66	75.0	33	67.3	33	84.6	
**Response to therapy**							
Excellent	71	80.7	35	71.4	36	92.3	0.0102
Poor	17	19.3	14	28.6	3	7.7	
**ATA risk category**							
Low	11	12.5	5	10.2	6	15.4	0.2361
Intermediate	32	36.4	15	30.6	17	43.6	
High	45	51.1	29	59.2	16	41.0	
**Post-operative thyroglobulin**							
Positive	32	38.1	22	44.9	10	28.6	0.1257
Negative	52	61.9	27	55.1	25	71.4	
**Hashimoto’s thyroiditis**							
Yes	24	27.3	12	24.5	12	30.8	0.5119
No	64	72.7	37	75.5	27	69.2	

### 3.4 Association of ATA risk categories with recurrence, persistent disease and response to therapy

In our cohort, ATA low-, intermediate- and high-risk categories were noted in 12.5% (11/88), 36.4% (32/88) and 51.1% (45/88), respectively. ATA high-risk group was found to be significantly associated with tumor recurrence (p = 0.0031) and poor response to therapy (p < 0.0001), but not with persistent disease (p = 0.8316) (**Table 5**).

**Table 5:** Association of ATA risk category with recurrence, persistence and response to therapy in pediatric DTC.

Low	Intermediate	High	p value
**Recurrence**				
Yes	0	5 (15.6%)	17 (37.8%)	0.0031
No	11 (100.0%)	27 (84.4%)	28 (62.2%)	
**Persistent disease**				
Yes	1 (9.1%)	4 (12.5%)	7 (15.6%)	0.8316
No	10 (81.8%)	28 (87.5%)	38 (84.4%)	
**Response to therapy**				
Excellent	11 (100.0%)	32 (100.0%)	28 (62.2%)	< 0.0001
Poor	0	0	17 (37.8%)	

## 4 Discussion

In this retrospective study of pediatric DTC patients, we have confirmed that DTC in children and adolescents present with advanced and more aggressive disease. In this cohort, pediatric DTC had a high incidence of LNM and distant metastasis (70.5% and 13.6%, respectively) as well as high rate of events (10 years EFS of 79.2%). This is consistent with several previous studies (8, 12, 22–25). Despite the presence of advanced disease in children with DTC in this cohort, long-term prognosis was excellent and 10 years overall survival was 98.4%, which is also in line with previous reports on pediatric DTC from different ethnicities (8, 13, 24, 26, 27).

To further determine potential independent factors for patients’ outcome, we included the clinico-pathological factors in multivariate Cox regression analysis. In order to determine the exact threshold for tumor size, we divided the patients by tumor size into different subgroups (<2cm, 2-3cm, 3-4cm and >4cm). Higher risk of DTC event free survival was observed in tumors larger than 4cm in both univariate and multivariate analysis. Hence, this size threshold was used for further analysis. Our results indicated that two predictive factors significantly and negatively impacted EFS: tumor size >4cm and the presence of initial distant metastasis. There are several reports where large tumor size and initial distant metastasis are found to have adverse impact on the recurrence and prognosis of pediatric DTC (14, 24, 28, 29). Although several other factors have been previously reported to be negatively associated with EFS, such as age, extent of resection and serum thyroglobulin levels (8, 25, 30), in our study, age, ATA risk stratification and other clinico-pathological parameters (extrathyroidal extension, bilaterality, multifocality, lymphovascular invasion and response to therapy) were not independent risk factors for EFS.

Our data shows trend towards association of younger age (<15 years) and EFS but this did not reach statistical significance (p = 0.0816), while several previous reports showed positive correlation between younger patients and worse prognosis (24, 30–35). Despite the lack of significant correlation between age and EFS in this cohort, younger patients did present with adverse clinico-pathological characteristics such as male gender, large tumor size, distant metastasis and poor response to therapy. Interestingly, only 71% of patients aged <15 years achieved complete remission whereas 92% patient aged >15 achieved complete remission (p = 0.0102).

In the recent ATA guidelines for adult DTC patients, risk stratification is clearly defined (17). However, whether ATA risk stratification effectively defines pediatric patients at risk of recurrent or persistent disease is unclear. Our data from pediatric DTC who underwent surgery and RAI shows that ATA risk stratification system effectively defines patient at risk of recurrence and response to therapy (**Table 5**) but does not correlate with persistent disease nor predicts EFS. This is contrary to recently published data (8, 36), where ATA risk group were identified as prognostic factor for EFS (15, 16). Whether this inconsistency is due to sample size or ethnic differences between the cohorts needs to be clarified by larger studies of pediatric DTC.

To explore if there are known genetic alteration that could affect the course of the disease in our cohort, we have analyzed *BRAF* and *TERT* mutation and found that *BRAF* mutation had no effect on patient’s outcome, whereas no *TERT* mutations were detected in our cohort.

Being retrospective in nature, this study has its inherent limitation, such as selection bias. Another limitation was that all patients included in this study underwent surgery and RAI. Our results should be interrupted with caution due to limited sample size and unique ethnicity.

In conclusion, our results suggest evaluating tumor size as a prognostic factor and risk stratification marker in pediatric DTC. Future studies are needed to confirm the impact of tumor size to define the likelihood of poor EFS and to guide risk adapted therapy and follow-up.

## Data availability statement

The original contributions presented in the study are included in the article/supplementary material. Further inquiries can be directed to the corresponding author.

## Ethics statement

The studies involving human participants were reviewed and approved by Research Advisory Council, King Faisal Specialist Hospital and Research Centre. Written informed consent for participation was not provided by the participants’ legal guardians/next of kin because: Since only retrospective patient data were used, waiver of written consent was granted.

## Author contributions

Study concept and design: KA-K, SP, AS. Executed the study: SP, AS, PA, NS, WH, SA-S, FA-D. Statistical analysis: SP. Drafting the article: KA-K, AS, SP. Critical revision of the article for important intellectual content, writing of the article, and approval of the final version: KA-K, SP, AS, PA, NS, WH, SA-S, FA-D. All authors contributed to the article and approved the submitted version.

## Acknowledgments

The authors would like to thank Felisa DeVera for and Kaleem Iqbal for their technical assistance.

## Conflict of interest

The authors declare that the research was conducted in the absence of any commercial or financial relationships that could be construed as a potential conflict of interest.

## Publisher’s note

All claims expressed in this article are solely those of the authors and do not necessarily represent those of their affiliated organizations, or those of the publisher, the editors and the reviewers. Any product that may be evaluated in this article, or claim that may be made by its manufacturer, is not guaranteed or endorsed by the publisher.
